# Assessment of left ventricular myocardial systolic dysfunction in premature ovarian insufficiency patients using echocardiographic layer-specific myocardial strain imaging

**DOI:** 10.1186/s44156-024-00056-x

**Published:** 2024-09-02

**Authors:** Yu-Lin Wang, Li-Xue Yin, Mei Li

**Affiliations:** 1 Ultrasound in Cardiac Electrophysiology and Biomechanics Key Laboratory of Sichuan Province, Sichuan Provincial People’s Hospital, University of Electronic Science and Technology of China, Chengdu, 610072 China; 2 Department of Gynaecology and Obstetrics, Sichuan Provincial People’s Hospital, University of Electronic Science and Technology of China, Chengdu, China

**Keywords:** Premature ovarian insufficiency, Layer-specific myocardial strain, Echocardiography, Left ventricle, Systolic function

## Abstract

**Background:**

Due to the lack of oestrogen, premature ovarian insufficiency (POI) is an independent risk factor for ischaemic heart disease and overall cardiovascular disease. This study aimed to apply layer-specific myocardial strain for early quantitative evaluation of subclinical left ventricular myocardial systolic function changes in patients with POI.

**Methods:**

Forty-eight newly diagnosed, untreated patients with POI (POI group) and fifty healthy female subjects matched for age, height and weight (control group) were enrolled. Standard transthoracic echocardiography was used to measure conventional parameters and layer-specific strain parameters.The layer-specific strain parameters included subendomyocardial global longitudinal strain (GLSendo), mid-layer myocardial global longitudinal strain (GLSmid), subepimyocardial global longitudinal strain (GLSepi), subendomyocardial global circumferential strain (GCSendo), mid-layer myocardial global circumferential strain (GCSmid), and subepimyocardial global circumferential strain (GCSepi).

**Results:**

There were no significant differences in age, body mass index (BMI), blood pressure, or left ventricular ejection fraction (LVEF) between the two groups. The end-diastolic interventricular septal thickness (IVST) was greater in the POI group (8.29 ± 1.32 vs. 7.66 ± 0.82, *P* = 0.008), and the POI group had lower E, E/A, and lateral e′ (all *P* < 0.05). As for systolic functions,the POI group had lower GLSendo, GLSmid, GLSepi, GCSendo, GCSmid, and GCSepi (all *P* < 0.05).The intraobserver and interobserver coefficients of GLSendo, GLSmid, GLSepi, GCSendo, GCSmid, and GCSepi were greater than 0.900.

**Conclusions:**

POI patients with normal LVEF may suffer from subclinical left ventricular myocardial systolic dysfunction. Echocardiography of layer-specific myocardial strain could more sensitively detect subclinical impairment of left ventricular systolic function in POI patients.

**Graphical abstract:**

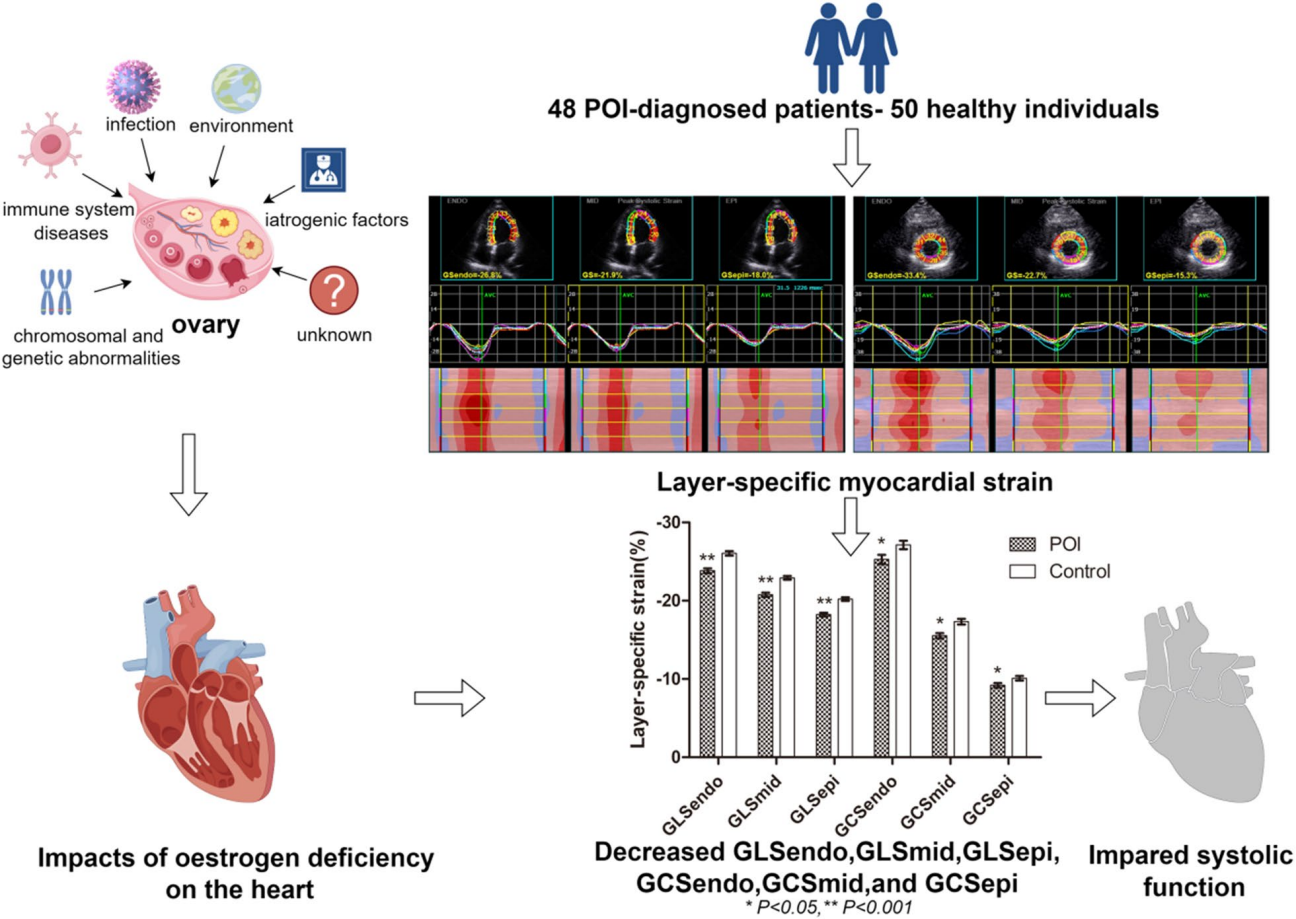

## Introduction

Premature ovarian insufficiency (POI) refers to a clinical syndrome caused by the loss of ovarian activity in women before the age of 40 years and mainly manifests as menstrual abnormalities (oligomenorrhoea or amenorrhoea), increased gonadotropins such as follicle-stimulating hormone (FSH) > 25 IU/L, and decreased oestrogen [1].

The global incidence rate of POI is 3.7% [2]. POI can be caused by chromosomal abnormalities and genetic defects (sex chromosomes and autosomes), immune system diseases, infection, or environmental or iatrogenic factors, but over 50% of patients with POI still have unknown aetiologies [3].

POI can have adverse effects on multiple systems in women, including the cardiovascular system, genitourinary system, skeletal system and mental health, and harm to the cardiovascular system can reduce life expectancy and increase the risk of heart failure [1, 4]. POI can serve as an independent risk factor for ischaemic heart disease (IHD) and cardiovascular disease (CVD) [5],and the pathophysiological mechanism affecting the cardiovascular system is mainly a series of changes caused by a lack of oestrogen. Most patients with POI have no obvious precordial discomfort in the early stage, so they often ignore changes in the cardiovascular system. In 2016, the European Society of Human Reproduction and Embryology (ESHER) recommended that all women with POI be assessed for CVD risk and receive early hormone replacement therapy (HRT) to control their future CVD risk [1]. The North American Menopausal Association also recommended HRT treatment for patients with POI without contraindications [4]. Turner syndrome (TS) is a special type of POI characterized by sex chromosomal abnormalities, and CVD-related risk factors should be assessed annually [1].

Layer-specific myocardial strain measurements are based on two-dimensional speckle tracking echocardiography (2D-STE), which can quantify the myocardial strain parameters of each layer, provide more accurate myocardial deformation information, and provide more guidance for clinical precision treatment. This study intended to use layer-specific myocardial strain to quantitatively assess subclinical myocardial systolic impairment of the left ventricle in patients with POI.

## Methods

Forty-eight patients who were newly diagnosed with POI from the Menopausal Gynecological Endocrinology Clinic of our hospital were included as the POI group.

All subjects with POI underwent sex hormone testing, and patients suspected of having TS underwent chromosome testing. Inclusion criteria: meeting the POI diagnostic criteria proposed by ESHER [1]: ① age < 40 years old; ② oligomenorrhoea or amenorrhoea for at least 4 months and elevated FSH > 25 IU/L on two occasions > 4 weeks apart. Exclusion criteria: ① age > 40 years old; ② prior HRT; ③ severe cardiovascular disease, including congenital cardiovascular disease, coronary heart disease, cardiomyopathy, severe arrhythmia, heart failure, pericardial disease; ④ hypertension, diabetes, blood system disease, autoimmune system disease, or history of radiotherapy or chemotherapy; and ⑤ poor image quality. Fifty healthy voluntary subjects matched for age, height and weight with the POI group were selected as the control group. These individuals had a normal menstruation history, sinus rhythm, no hypertension, no hyperlipidaemia, no diabetes, and no history of severe cardiovascular disease. This study was reviewed and approved by the hospital ethics committee, and all the subjects signed informed consent.

Blood pressure was measured by a cuff manometer for all subjects.Complete echocardiography was performed by using Vivid E9 echocardiographic diagnostic equipment (GE Healthcare) with an M5S probe (frequency: 1.4–4.6 MHz). Two-dimensional dynamic images of at least three cardiac cycles were collected from the parasternal left ventricle long-axis view, apical 4-chamber view, apical 3-chamber view, apical 2-chamber view, left ventricular mitral valve short-axis view, papillary muscle short-axis view, and apical short-axis view. Pulsed-wave Doppler (PW) was used to collect anterior blood flow spectrum of the mitral valve in the apical 4-chamber view, and tissue Doppler imaging (TDI) was subsequently used to obtain the velocity spectrum of the lateral and septal mitral annulus.

A dedicated EHCO PAC 202 workstation was used for echocardiographic image postprocessing and measurement of the end-diastolic diameter of the left ventricle (LVEDD), the end-diastolic thickness of the left ventricular posterior wall (LVPWT), and the end-diastolic interventricular septal thickness (IVST) in the parasternal left ventricular long-axis view. The following formulas were used to calculate relative wall thickness (RWT) = 2 × LVPWT/LVEDD, left ventricular mass (LVM) = 0.8 × 1.04 × [(LVEDD + IVST + LVPWT)^3^ − LVEDD^3^] + 0.6, body surface area (BSA) = 0.0061 × height (cm) + 0.0128 × weight (kg) − 0.1529, body mass index (BMI) = weight (kg)/height(m)^2^, and left ventricular mass index (LVMI) = LVM/BSA. Left ventricular ejection fraction (LVEF) was measured by the biplane Simpson method, the left atrial volume (LAV) was measured by the biplane Simpson method at the apical 4- and 2-chamber views, and the left ventricular volume index (LAVI) was calculated as LAV/BSA [6].

**Fig. 1:**
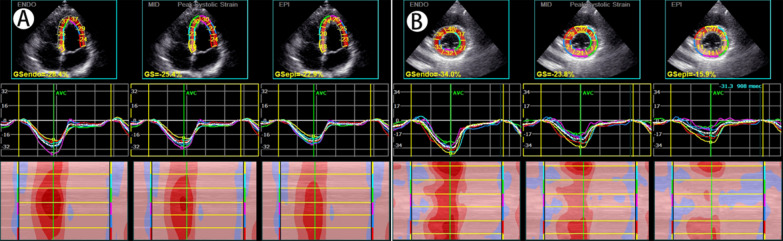
The software automatically tracks the movement trajectory of the myocardial spots in each layer to obtain layer-specific strain parameters. **A** Shows a schematic diagram of the apical 4-chamber view, and **B** shows a schematic diagram of the left ventricular mitral valve short-axis view

The apical 3-chamber view, apical 2-chamber view, apical 4-chamber view, left ventricular mitral valve short-axis view, left ventricular papillary muscle short-axis view, and left ventricular apical short-axis view were selected. The Q-analysis system was started, the 2D-strain was entered to manually trace the boundary of the endocardium and epicardium, the region of interest (ROI) was determined, and the software automatically tracked the movement trajectory of the myocardial spots in each layer. Finally, the subendomyocardial global longitudinal strain (GLSendo), the mid-layer myocardial global longitudinal strain (GLSmid), the subepimyocardial global longitudinal strain (GLSepi), the subendomyocardial global circumferential strain (GCSendo), the mid-layer myocardial global circumferential strain (GCSmid), and the subepimyocardial global circumferential strain (GCSepi) were obtained (Fig. 1).

The evaluation of left ventricular diastolic function was based on guidelines proposed by ASE/EACI in 2016 [7]. Using a simple random sampling method, 10 patients were randomly selected among the POI patients for intraobserver reproducibility testing, and another 10 patients were randomly selected for interobserver reproducibility testing.

SPSS 25.0 was used for statistical analysis. The data are expressed as mean ± standard deviation. The Kolmogorov–Smirnov test was used to determine the distribution type of continuous variables, and the Levene test was used to test the homogeneity of variance. If the data from both groups satisfied both the normal distribution and homogeneity of variance criteria, the two-independent-samples t test was performed; otherwise, the Mann–Whitney U test was used for two independent samples. The intraclass correlation coefficient (ICC) was used for the reproducibility test. *P* < 0.05 was statistically significant for all analyses.

## Results

Among the forty-eight patients in the POI group, the oestrogen levels of twenty-two patients were less than 37 pmol/L (the lowest level of serum sex hormone testing equipment was 37 pmol/L), the oestrogen levels of the other twenty-six patients were 88.37 ± 48.68 pmol/L, and the FSH level of the POI group was 66.70 ± 27.89 mIU/ml. There were five patients with TS in the POI group; three of these patients had 45,X, and the chromosomes of the other two patients were 45,X/46,XX/47,XXX and 45,X/46,X,r(X). Among the POI patients, four had primary amenorrhoea, and the abnormal menstruation history of the other forty-four patients was 13.07 ± 9.33 months.

No significant difference in age, BSA, BMI, systolic blood pressure, diastolic blood pressure, or heart rate was found between the two groups (all *P* > 0.05) (Table 1).

**Table 1:** Routine clinical data of the two groups

Variable	POI (n = 48)	Control (n = 50)	P value
Age (years)	33.00 ± 6.27	31.64 ± 7.34	0.073
BSA (m^2^)	1.50 ± 0.13	1.51 ± 0.10	0.741
BMI (kg/m^2^)	21.15 ± 2.45	20.59 ± 2.24	0.239
Systolic BP (mmHg)	112.44 ± 10.47	112.96 ± 9.56	0.797
Diastolic BP (mmHg)	69.79 ± 9.46	69.54 ± 7.27	0.878
Heart rate (bpm)	75.48 ± 9.81	73.08 ± 8.21	0.134

*BSA *body surface area, *BMI* body mass index, *BP* blood pressure

The IVST in the POI group was greater than that in the control group (*P* = 0.008), but there were no significant differences between the two groups in LVPWT, LVEDD, RWT, LVM, or LVMI (all *P* > 0.05). The values of E, E/A, and lateral e′ in the POI group were lower than those in the control group (all* P* < 0.05), while no differences in A, septal e′, average E/e′ or LVEF were found between the two groups (*P* > 0.05) (Table 2).

**Table 2:** Conventional echocardiographic parameters of the two groups

Variable	POI (n = 48)	Control (n = 50)	P value
IVST (mm)	8.29 ± 1.32	7.66 ± 0.82	0.008
LVPWT (mm)	7.73 ± 1.35	7.49 ± 1.04	0.468
LVEDD (mm)	40.79 ± 3.22	41.85 ± 3.24	0.108
RWT	0.38 ± 0.08	0.36 ± 0.06	0.130
LVM (g)	97.30 ± 18.57	94.72 ± 17.46	0.352
LVMI (g/m^2^)	64.76 ± 11.39	62.82 ± 11.54	0.233
E (m/s)	0.83 ± 0.13	0.94 ± 0.14	0.000
A (m/s)	0.63 ± 0.16	0.60 ± 0.15	0.378
E/A	1.53 ± 0.57	1.67 ± 0.50	0.038
Lateral e′ (m/s)	0.14 ± 0.02	0.15 ± 0.03	0.004
Septal e′ (m/s)	0.12 ± 0.03	0.13 ± 0.04	0.073
Average E/e′	6.80 ± 1.64	7.01 ± 1.86	0.782
LAVI (mL/m^2^)	21.49 ± 5.84	21.13 ± 5.74	0.761
LVEF (%)	65.56 ± 6.05	66.78 ± 5.48	0.545

*IVST* end-diastolic interventricular septal thickness, *LVPWT*  end-diastolic thickness of left ventricular posterior wall, *LVEDD* end-diastolic diameter of left ventricle, *RWT* relative wall thickness, *LVM* left ventricular mass, *LVMI* left ventricular mass index, *E* early diastolic peak velocity of mitral flow, *A* late diastolic peak velocity of mitral flow, *E/A* the ratio of E to A, *lateral e′* early diastolic peak velocity of mitral lateral annulus, *septal* *e′* early diastolic peak velocity of mitral septal annulus, *Average E/e′* the ratio of E to average e′, *LAVI* left atrial volume index, *LVEF* left ventricular ejection fraction

As assessed under established guidelines [7], the left ventricular diastolic function of all the subjects remained normal, but the presence of individual abnormalities in the POI group (E, E/A, lateral e′) still suggested that there were adverse changes in left ventricular diastolic function in POI patients.

**Fig. 2:**
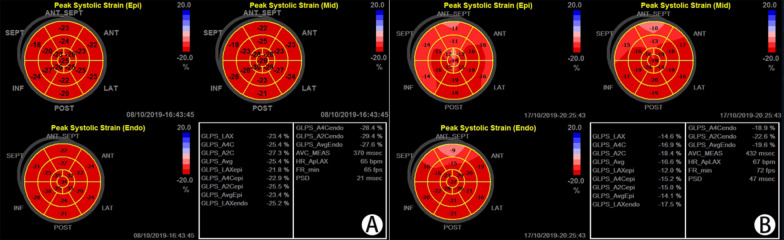
Comparison of global longitudinal layer-specific strains and bull's eye diagrams in peak systolic between the control group and the POI group. **A** Shows the GLSendo, GLSmid, GLSepi and bull's eye diagrams in the control group, and **B** shows the same in the POI group. Each segment in the bull's eye diagram in **A** was uniformly red, but some segments in **B** became lighter and uneven, indicating the strains of these segments decrease

**Fig. 3:**
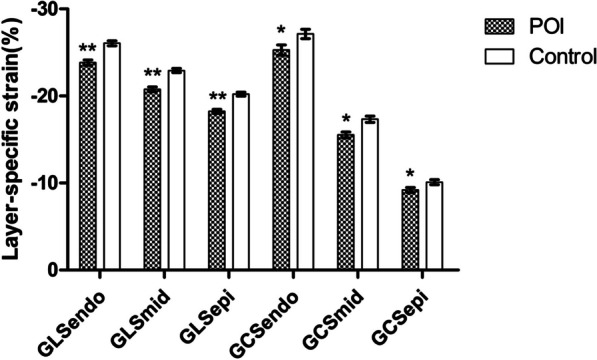
Comparison of longitudinal layer-specific strains and circumferential layer-specific strains between the POI group and control group. GLSendo, GLSmid, GLSepi, GCSendo, GCSmid and GCSepi were all lower in the POI group. Similarly, the longitudinal and circumferential strain parameters of the two groups all exhibited a decreasing trend from the subendocardium to the subepicardium.*P < 0.05, **P < 0.001. Standard error was the measure of sampling error

GLSendo, GLSmid, GLSepi, GCSendo, GCSmid and GCSepi were all lower in the POI group (*P* < 0.05 or *P* < 0.001). The longitudinal strain and circumferential strain parameters of the two groups all showed a decreasing trend from the subendocardium to the subepicardium (Table 3) (Figs. 2, 3).

**Table 3:** Layer-specific strains of the two groups

Variable	POI (n = 48)	Control (n = 50)	P value
GLSendo (%)	− 23.81 ± 3.76	− 26.03 ± 3.62	0.000
GLSmid (%)	− 20.74 ± 3.29	− 22.90 ± 3.00	0.000
GLSepi (%)	− 18.20 ± 2.96	− 20.20 ± 2.75	0.000
GCSendo (%)	− 25.26 ± 7.12	− 27.10 ± 6.66	0.023
GCSmid (%)	− 15.50 ± 4.10	− 17.31 ± 4.48	0.001
GCSepi (%)	− 9.18 ± 3.41	− 10.07 ± 3.43	0.047

*GLSendo* subendomyocardial global longitudinal strain, *GLSmid* mid-layer myocardial global longitudinal strain, *GLSepi* subepimyocardial global longitudinal strain, *GCSendo* subendomyocardial global circumferential strain, *GCSmid* mid-layer myocardial global circumferential strain, *GCSepi* subepimyocardial global circumferential strain

The intraobserver and interobserver coefficients of GLSendo, GLSmid, GLSepi, GCSendo, GCSmid, and GCSepi were greater than 0.900, so the layer-specific strains had acceptable reproducibility (Table 4).

**Table 4:** Intraobserver and interobserver reproducibility of layer-specific stains

Variable	Intraobserver coefficient	95% Confidence level	Interobserver coefficient	95% Confidence level
GLSendo (%)	0.942	0.881–0.972	0.936	0.871–0.969
GLSmid (%)	0.941	0.880–0.971	0.929	0.858–0.966
GLSepi (%)	0.915	0.830–0.958	0.920	0.840–0.961
GCSendo (%)	0.957	0.912–0.979	0.941	0.881–0.971
GCSmid (%)	0.967	0.932–0.984	0.953	0.902–0.978
GCSepi (%)	0.957	0.908–0.979	0.938	0.872–0.970

*GLSendo* subendomyocardial global longitudinal strain, *GLSmid* mid-layer myocardial global longitudinal strain, *GLSepi* subepimyocardial global longitudinal strain, *GCSendo* subendomyocardial global circumferential strain, *GCSmid* mid-layer myocardial global circumferential strain, *GCSepi* subepimyocardial global circumferential strain

## Discussion

Due to the loss of functional ovarian follicles, women with POI constantly have low oestrogen and face infertility, perimenopausal syndrome and other problems. Patients with POI also have a higher CVD risk [8–10], and early menopause is associated with an increased risk of heart failure [11].Since most women with POI do not have chest tightness, chest pain or other CVD-related clinical symptoms in the early stage, the risk of CVD is often ignored. Serological indicators and blood pressure are often used to detect the risk of CVD in patients with POI, but echocardiography is rarely used to test the myocardial function of POI patients. Layer-specific myocardial strain can more sensitively reflect subclinical myocardial changes in the early stage of disease than LVEF can [12, 13] and has been used in many recent studies of various diseases [14–16]. Therefore, we aimed to use layer-specific myocardial strain to evaluate left ventricular myocardial systolic function in POI patients.

In this study, the POI group had a thicker IVST than the control group (*P* = 0.008), suggesting that patients with POI may have left ventricular remodelling. According to the criteria for left ventricular hypertrophy proposed in guidelines [6] (LVMI > 95 g/m^2^ in females), all subjects in this study did not meet the criteria for left ventricular hypertrophy, but fifteen patients (34.25%) in the POI group met the criteria for left ventricular concentric remodelling (RWT > 0.42, LVMI ≤ 95 g/m^2^), indicating the occurrence of left ventricular remodelling in patients with POI. The possible pathophysiological mechanisms include the following: Oestradiol (E_2_), as a fast activator of endothelial nitric oxide synthase (NOS),can stimulate the production of nitric oxide (NO), and a lack of E_2_ in patients with POI results in reduced NO production by vascular endothelial cells and decreased vascular elasticity and compliance,then cardiac afterload increases, which promotes left ventricular remodelling and myocardial hypertrophy [17, 18]; otherwise, a low E_2_ can lead to abnormalities in the renin-angiotensin system (RAS) in the heart and systemic circulation and enhance the activity of angiotensin-converting enzyme (ACE), leading to ventricular remodelling and cardiac hypertrophy [19].

Echocardiography can not only evaluate changes in left ventricular geometry but also quantitatively evaluate changes in function. According to the guideline [7], apparent decreases in left ventricular diastolic function were not detected in any of the subjects; however, the POI group had lower E, E/A, and lateral e′ (*P* < 0.05 or *P* < 0.01), indicating there are still some signs of decreased diastolic function, which is generally consistent with relevant research [20]. An animal experiment showed that the rMCP-1 and rMCP-5 mRNAs in the cardiomyocytes of ovariectomized rats, as the isoforms of chymase that form angiotensin II (Ang II), participate in the loss of normal heart function and structure caused by ovariectomy in rodents, as indicated by decreased left ventricular diastolic function (increased E/e’) and increased LVEDD [21]. Another study showed deficiency of E_2_ led to reduced mitochondrial function in cardiomyocytes and inhibited NOS signalling, thereby inhibiting the GPR30 pathway, which together led to a decreased left ventricular diastolic function [18].

Changes in left ventricular diastolic function are often inseparable from changes in systolic function. However, there was no obvious abnormality in LVEF (*P* > 0.05), but conventional LVEF often cannot reflect subclinical changes in left ventricular systolic function. This study used layer-specific myocardial strain and revealed that GLSendo, GLSmid, GLSepi, GCSendo, GCSmid, and GCSepi in the POI group were lower than those in the control group. There findings suggested that there was a reduction in left ventricular systolic function in the POI group and that layer-specific myocardial strain could more sensitively detect left ventricular systolic function impairment in patients with POI than two-dimensional LVEF. Some possible mechanisms for the decrease in layer-specific strains of the left ventricle in the POI group are as follows: An animal experiment showed that E_2_ enhanced cardiac systolic function by regulating the expression of cAMP-L-type Ca_2_^+^ channel (cAMP-LTCC)-related genes and promoted cardioprotection against stress through the GPR30 nongenomic acute signalling pathway [22]. In addition, there are oestrogen receptors (ERs) in the arterial wall and myocardial tissue, including ERα, ERβ, and the G protein-coupled oestrogen receptor (GPER), and E_2_ might directly act on cardiomyocytes to enhance myocardial systolic function [23–25].There is a certain gradient in the deformability of the normal left ventricle myocardium, and the deformation of the myocardium from the endocardium to epicardium gradually decreases [26]. This study also confirmed this decreasing trend from the endocardium to the epicardium in all subjects.

Limitations of this study: The hospital serum sex hormone test equipment had 37 pmol/L as the baseline for oestrogen, so we could not collect the E_2_ levels of patients whose E_2_ levels were lower than 37 pmol/L. The sample was fairly small. This was only a preliminary study, and a follow-up study during hormone replacement therapy (HRT) was not conducted.

In summary, POI patients with normal LVEF may have subclinical left ventricular myocardial systolic impairment. Layer-specific myocardial strain can more sensitively detect subclinical left ventricular systolic impairment in patients with POI.

## Data Availability

The data underlying this article will be shared on reasonable request to the corresponding author.
